# Case Report: Whole genome sequencing identifies a novel deep intronic *COL4A5* variant of uncertain significance in X-linked Alport syndrome

**DOI:** 10.3389/fped.2025.1639471

**Published:** 2025-08-07

**Authors:** Hoon Seok Kim, Myungshin Kim, Jin-Soon Suh, Yeonhee Lee

**Affiliations:** ^1^ Department of Laboratory Medicine, Seoul St. Mary’s Hospital, College of Medicine, The Catholic University of Korea, Seoul, Republic of Korea; ^2^ Catholic Genetic Laboratory Center, Seoul St. Mary’s Hospital, College of Medicine, The Catholic University of Korea, Seoul, Republic of Korea; ^3^ Department of Pediatrics, Bucheon St. Mary’s Hospital, College of Medicine, The Catholic University of Korea, Seoul, Republic of Korea; ^4^ Department of Pediatrics, Seoul St. Mary’s Hospital, College of Medicine, The Catholic University of Korea, Seoul, Republic of Korea

**Keywords:** alport syndrome, *COL4A5*, deep intronic variant, whole genome sequencing, pediatric nephrology

## Abstract

Diagnosing Alport syndrome can be particularly challenging when targeted sequencing methods, such as panel-based next-generation sequencing (NGS), fail to identify pathogenic variants, especially deep intronic mutations. The syndrome is caused by mutations in type IV collagen genes (*COL4A3*, *COL4A4*, or *COL4A5*), with X-linked Alport syndrome (XLAS) accounting for approximately 80% of cases. Here, we report the case of a 4-year-old boy who presented with persistent microscopic hematuria detected during routine urinalysis. Although renal ultrasonography showed mild bilateral medullary nephrocalcinosis, no proteinuria was observed. His mother had been previously diagnosed with Alport syndrome by renal biopsy, but prior targeted sequencing failed to identify any disease-causing variants. To avoid an invasive renal biopsy in this pediatric patient, we directly performed whole genome sequencing (WGS), identifying a novel deep intronic hemizygous variant in the *COL4A5* gene (c.2395 + 2723T > G). This variant, classified as a variant of uncertain significance (VUS) according to ACMG-AMP guidelines, was confirmed by Sanger sequencing to be hemizygous in the patient and heterozygous in his mother. The patient currently maintains normal renal function, vision, and hearing, with only microscopic hematuria persisting. This case highlights the diagnostic challenges posed by deep intronic variants in XLAS and demonstrates the clinical utility of WGS in cases where conventional genetic testing is inconclusive. Early genetic diagnosis facilitated timely intervention without requiring invasive procedures, emphasizing the growing role of comprehensive genomic sequencing in uncovering elusive genetic variants in clinically suspected Alport syndrome.

## Introduction

Alport syndrome is an inherited kidney disorder characterized by progressive glomerular disease, often accompanied by sensorineural hearing loss and ocular abnormalities (1). This rare genetic condition affects approximately 1 in 50,000 individuals across all ethnicities and populations, representing the second most common monogenic kidney disease and accounting for 0.5% of adult and 1.7% of pediatric end-stage kidney disease cases in the United States (2).

First reported by Arthur Cecil Alport in 1927 as “Hereditary Familial Congenital Hemorrhagic Nephritis,” the diagnostic criteria for Alport syndrome have evolved over time (3). Historically, strict clinical criteria were required for diagnosis, including characteristic renal biopsy findings, sensorineural hearing loss, ocular abnormalities, and persistent hematuria and renal dysfunction, often accompanied by a relevant family history (4, 5). However, recent expert consensus guidelines recommend genetic confirmation as essential, defining Alport syndrome more broadly to include all individuals harboring pathogenic variants in *COL4A3*, *COL4A4*, or *COL4A5*, regardless of their clinical presentation (6–8). Although molecular technologies, such as direct sequencing and targeted panel-based next-generation sequencing (NGS), have become increasingly utilized due to their diagnostic reliability and non-invasiveness, these methods have limitations. Notably, they typically do not detect variants located in deep intronic or regulatory regions, potentially resulting in missed diagnoses (9–12).

Genetically, Alport syndrome is caused by pathogenic variants in type IV collagen genes (*COL4A3*, *COL4A4*, and *COL4A5*), which lead to abnormalities in the glomerular basement membrane resulting in kidney dysfunction and progressive renal failure (13, 14). The condition follows four primary inheritance patterns, with X-linked Alport syndrome (XLAS) being the most common, accounting for approximately 80% of cases due to mutations in the *COL4A5* gene (15). XLAS primarily affects males, while female carriers are at risk of developing hematuria and renal failure. The remaining cases consist of autosomal recessive Alport syndrome (ARAS, 15%), autosomal dominant Alport syndrome (ADAS, 5%), and, very rarely, digenic inheritance (16, 17). While molecular genetic testing is recommended when Alport syndrome is clinically suspected, cost and accessibility considerations necessitate careful selection of testing methods and timing.

Importantly, emerging evidence indicates that deep intronic variants, which create cryptic splice sites or cause pseudo-exon inclusion, may account for cases undiagnosed by conventional exome sequencing (12). This suggests the inherent limitations of conventional NGS panel sequencing or exome sequencing approaches, underscoring the increasing importance of whole genome sequencing (WGS) in clinical diagnostics (9, 18).

In this study, we present a pediatric case of XLAS, where a novel deep intronic variant in the *COL4A5* gene was successfully identified using WGS, underscoring the diagnostic complexities and clinical implications of such elusive genetic alterations.

## Case description

**Figure 1:**
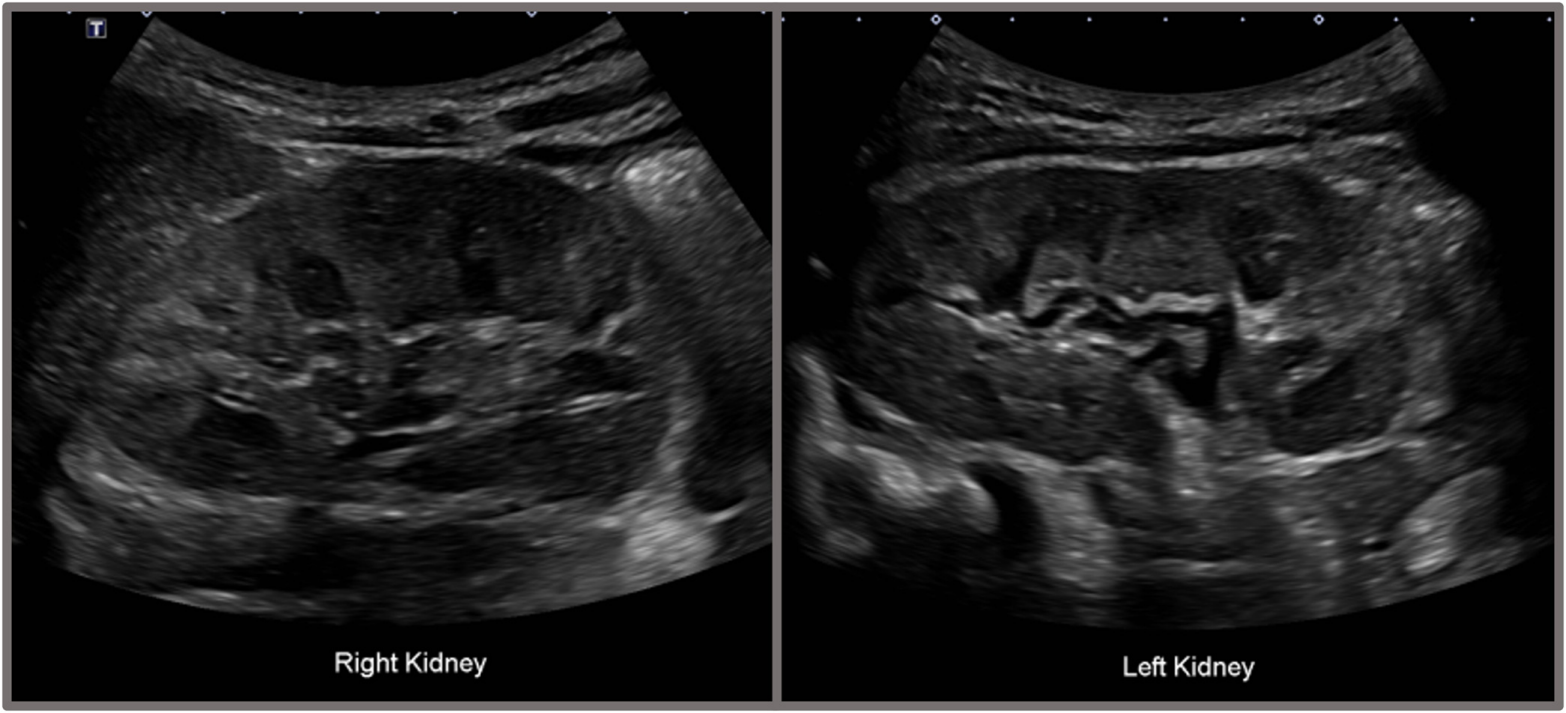
Renal ultrasonography of the patient showing bilateral grade I medullary nephrocalcinosis. Renal ultrasonography of the patient demonstrated bilateral grade I medullary nephrocalcinosis. The right kidney measured 7.8 × 3.7 cm, and the left kidney measured 7.3 × 3.8 cm, both within normal size limits. The cortical echogenicity of both kidneys was normal. Multiple hyperechogenic foci were observed in the medullary regions of both kidneys, and some medullary pyramids exhibited partial hyperechoic rims.

A 4-year-old boy presented to our outpatient clinic with persistent microscopic hematuria initially detected during routine health screening urinalysis. At first, the hematuria was presumed to be due to cystitis, leading to an initial antibiotic treatment. However, microscopic hematuria persisted despite the absence of pyuria or bacteriuria, prompting referral to the pediatric nephrology clinic for further evaluation. Clinical examination revealed no proteinuria, hearing impairment, or other noticeable symptoms. Laboratory evaluation revealed a hemoglobin level of 11.8 g/dl, white blood cell count of 11,350/μl, platelet count of 532,000/μl, blood urea nitrogen of 11.2 mg/dl, serum creatinine of 0.41 mg/dl (estimated GFR 106 ml/min/1.73 m²), serum albumin of 4.1 g/dl, and total cholesterol of 135 mg/dl. Urinalysis confirmed significant microscopic hematuria (RBC >100/HPF) with a urine calcium/creatinine ratio of 0.022 mg/mg. Renal ultrasound identified grade I medullary nephrocalcinosis bilaterally (Figure 1), though kidney size and structure remained normal.

**Figure 2:**
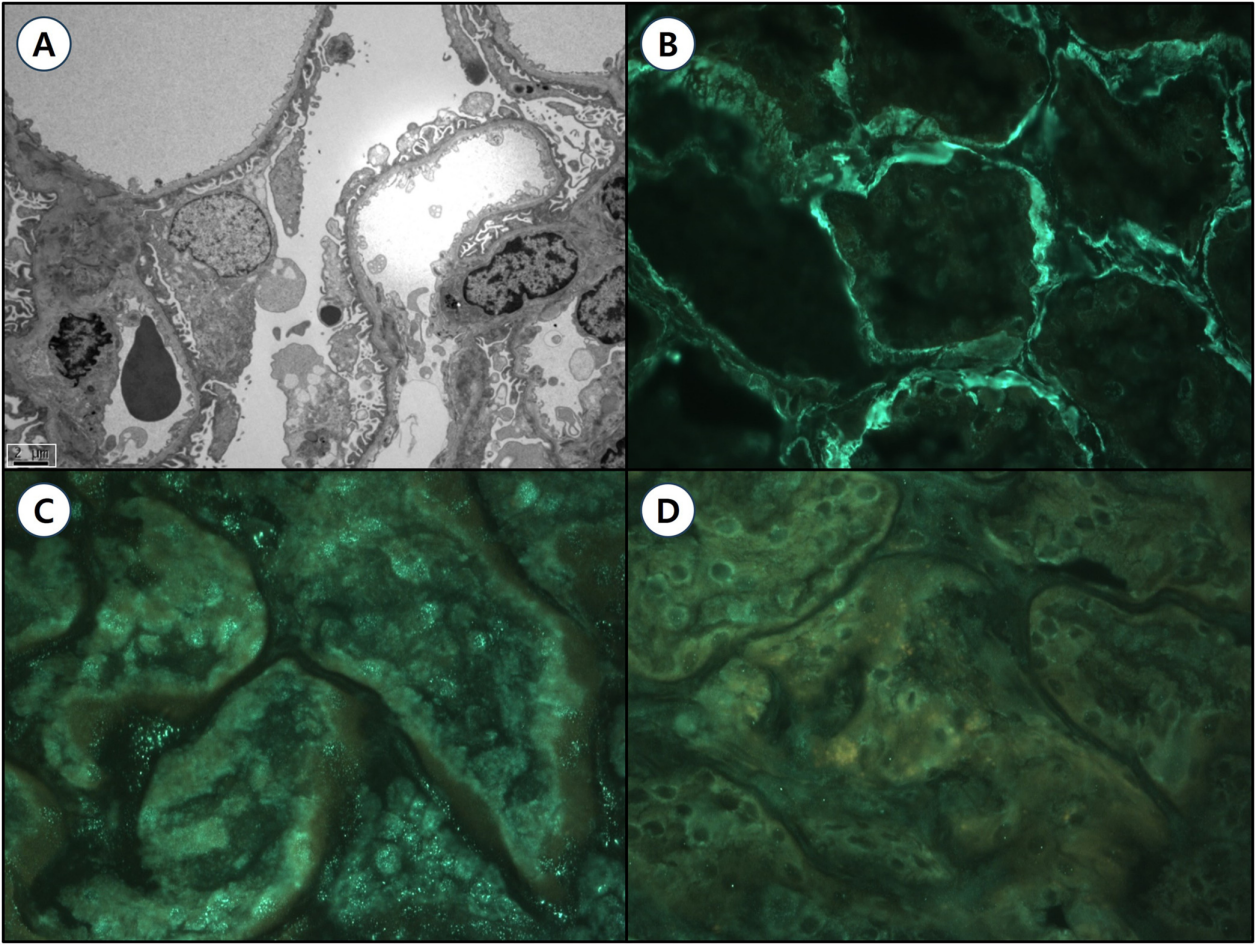
Renal pathological findings of the patient's mother with Alport syndrome. **(A)** Transmission electron microscopy (TEM, magnification ×4,000, scale bar = 2 μm) reveals ultrastructural abnormalities of the glomerular basement membranes (GBMs), characterized by alternating thinning and thickening (ranging from 150 to 1,045 nm; mean thickness 385 nm) compared to institutional reference values (296–481 nm). Moderate effacement of epithelial cell foot processes and focal rarefaction, degeneration, and granular material deposition were observed without definite lamellation. Mesangial basement membranes appear normal without significant deposits. (B–D) Immunofluorescence staining for type IV collagen α chains (magnification ×400): **(B)** α1 chain: Diffuse, strong linear positivity in both glomerular and tubular basement membranes (GBM 3+, TBM 3+). **(C)** α3 chain: Diffuse, weakly positive linear staining along the GBM (GBM 2+). **(D)** α5 chain: Negative staining in the GBM, consistent with X-linked Alport syndrome.

The patient's mother had been diagnosed with Alport syndrome approximately ten years earlier at the age of 25, following incidental detection of proteinuria and hematuria during routine screening. Her urinalysis at the time revealed red blood cells (RBCs) of 30–49/HPF, an albumin-to-creatinine ratio (ACR) of 332.77 mg/g, a protein-to-creatinine ratio (PCR) of 0.481 mg/g, and serum blood urea nitrogen (BUN)/creatinine levels of 15/0.69 mg/dl, corresponding to an estimated glomerular filtration rate (eGFR) of 103 ml/min/1.73 m². A renal biopsy demonstrated characteristic ultrastructural abnormalities on electron microscopy (Figure 2A), showing glomerular basement membranes (GBMs) with alternating thinning and thickening (150–1045 nm; mean 385 nm), compared to institutional reference values (296–481 nm). Immunofluorescence staining for type IV collagen α chains (α1, α3, and α5) showed diffuse linear expression of α1 and α3 chains, while the α5 chain was absent (Figures 2B–D). These findings established a definitive diagnosis of Alport syndrome in the patient's mother. However, her clinical exome sequencing including *COL4A3*, *COL4A4*, and *COL4A5* failed to identify pathogenic variants. The patient's father and paternal uncles exhibited no abnormalities on urinalysis, and their renal function was within normal limits.

**Figure 3:**
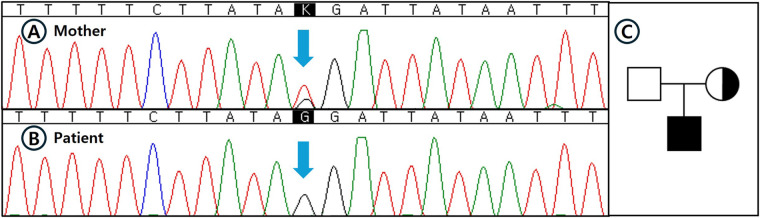
Sanger sequencing confirmation and pedigree analysis of the identified deep intronic *COL4A5* variant. **(A–B)** Sanger sequencing validation of the deep intronic *COL4A5* variant (NM_033380.3:c.2395 + 2723T > G) that was initially identified by whole genome sequencing (WGS): **(A)** The patient's mother showing a heterozygous state for the variant. **(B)** The patient demonstrating a hemizygous state for the variant. Blue arrows indicate the nucleotide substitution site. **(C)** Pedigree of the family affected by X-linked Alport syndrome (XLAS). Filled symbol indicates the patient (hemizygous), dotted circle represents the heterozygous mother, and open symbol indicates the unaffected father. The pedigree reflects confirmed genotypes based on sequencing results.

Given the persistent microscopic hematuria and strong family history, and in accordance with recent expert guidelines recommending genetic testing for persistent hematuria when targeted sequencing methods such as panel-based NGS fail to identify pathogenic variants, we opted for WGS instead of performing an invasive renal biopsy (6–8). WGS was conducted using genomic DNA extracted from peripheral blood, sequenced at an average depth of coverage of 32.45× on the Illumina NovaSeq 6000 platform, and processed using the EVIDENCE v4.1 pipeline (3billion, Seoul, Korea) based on Genome Reference Consortium Human Build 38 (GRCh38). A novel deep intronic hemizygous variant (NM_033380.3:c.2395 + 2723T > G) located at genomic position X-108609615-T-G (GRCh38), within intron 29 of the *COL4A5* gene, was identified. This variant was absent in public population databases, including gnomAD v3.1.2, ClinVar, and HGMD (meeting ACMG criterion PM2), and in silico splice-site prediction analysis using SpliceAI indicated a moderate probability of altering splicing (SpliceAI score: 0.47, meeting ACMG criterion PP3). Subsequent Sanger sequencing confirmed hemizygosity in the patient and heterozygosity in his mother, supporting its pathogenic potential related to XLAS (Figure 3) (meeting ACMG criterion PP1). Accordingly, this variant was classified as a variant of uncertain significance (VUS) based on ACMG-AMP criteria PM2, PP3, and PP1, incorporating the Alport syndrome-specific modifications of the ACMG standards and guidelines for the interpretation of sequence variants (19, 20). Ophthalmological and otolaryngological examinations confirmed normal vision and hearing at the current stage. Identifying this elusive genetic variant through comprehensive genomic analysis allowed for an early genetic diagnosis, thereby avoiding invasive procedures and facilitating timely clinical management and counseling.

## Discussion

This report describes a novel deep intronic variant (c.2395 + 2723T > G) in the *COL4A5* gene, identified in a pediatric patient presenting with persistent microscopic hematuria and a family history of kidney disease. This variant was detected through WGS and confirmed by Sanger sequencing, highlighting diagnostic challenges associated with deep intronic variants. Indeed, conventional genetic testing methods such as targeted panel sequencing or exome sequencing typically fail to detect these intronic variants due to their inherent limitations in capturing non-coding regions. Previous studies have reported that approximately 15% of X-linked Alport syndrome (XLAS) cases involve splicing mutations, including deep intronic variants that cause cryptic splice site activation or pseudo-exon inclusion, disrupting the coding sequence and impairing normal protein function (9–12, 18). Such deep intronic variants are increasingly recognized as a significant cause of genetically unresolved Alport syndrome, emphasizing the importance of genetic testing approaches capable of identifying these elusive mutations.

Alport syndrome arises from structural abnormalities in the type IV collagen network of the glomerular basement membrane (GBM) (21). Type IV collagen, composed of six distinct alpha chains (α1–α6) (22–24), is the primary structural component of basement membranes. Pathogenic variants in the *COL4A3*, *COL4A4*, or *COL4A5* genes disrupt the proper assembly and structure of collagen type IV, leading to impaired GBM integrity and progressive renal dysfunction, and may also affect ocular and cochlear structures (1, 24). Microscopic hematuria is the most common early symptom of XLAS, although some individuals remain asymptomatic. Importantly, males with XLAS typically progress to end-stage kidney disease within the first three decades of life, highlighting the necessity of timely diagnosis and intervention (25). Early therapeutic interventions, particularly renin-angiotensin-aldosterone system (RAAS) blockade, have demonstrated efficacy in slowing disease progression, underscoring the importance of early genetic diagnosis to facilitate prompt initiation of treatment (26). Nonetheless, the heterogeneity of clinical presentations and the limitations of standard genetic methods frequently contribute to delayed or missed diagnoses.

In this case, comprehensive genomic analysis using WGS uniquely facilitated the identification of a deep intronic *COL4A5* variant that conventional exome and targeted sequencing had previously failed to detect. The identified variant (c.2395 + 2723T > G) was classified as a VUS according to ACMG-AMP guidelines with disease-specific modifications, based on its absence from population databases (PM2), computational predictions indicating a potential splicing impact (PP3), and co-segregation evidence within the family (PP1) (19, 20). Similar deep intronic variants causing cryptic splice site activation and pseudo-exon inclusion have been previously reported in *COL4A5*, reinforcing the potential clinical significance of these alterations (9–12, 18). However, this study has an important limitation: functional validation studies, such as RNA sequencing or minigene assays, were not performed. Such analyses are essential to definitively confirm the splicing impact and the pathogenicity of deep intronic variants, and future functional investigations will be crucial for clarifying the clinical relevance of the identified variant.

Clinically, classifying variants as uncertain significance creates considerable challenges in medical decision-making, as uncertain pathogenicity necessitates careful clinical correlation and cautious genetic counseling. Regular monitoring of renal function and clinical evaluation of affected and at-risk family members are advisable to detect potential disease progression or to obtain additional evidence that could clarify the variant's clinical significance. Confirming the pathogenicity of this variant through subsequent functional studies may provide critical prognostic insights, enabling more precise, personalized management and genetic counseling. Furthermore, early genetic diagnosis has broader clinical implications beyond merely confirming the diagnosis. It facilitates personalized patient management, enabling timely implementation of interventions, such as RAAS blockade therapy, that can significantly delay kidney disease progression. Identifying the genetic cause also provides essential information for genetic counseling, allowing at-risk family members to undergo targeted genetic evaluation and receive appropriate monitoring and early therapeutic interventions, thereby potentially reducing the necessity for invasive diagnostic procedures such as renal biopsy. Consequently, comprehensive genomic sequencing approaches, particularly WGS, represent an essential tool in diagnosing genetically elusive cases of Alport syndrome and improving patient outcomes through early and accurate genetic diagnosis.

## Data Availability

The datasets presented in this article are not readily available because of ethical and privacy restrictions. Requests to access the datasets should be directed to the corresponding author.
